# Selective immunoglobulin M deficiency in a patient with celiac disease and recurrent pneumonia

**DOI:** 10.1002/ccr3.3489

**Published:** 2020-12-18

**Authors:** Marziyeh Heidarzadeh Arani, Mohsen Razavizadeh, Reza ArefNezhad, Hossein Motedayyen

**Affiliations:** ^1^ Department of Pediatrics Faculty of Medicine Kashan University of Medical Sciences Kashan Iran; ^2^ Research Center for Biochemistry and Nutrition in Metabolic Diseases Kashan University of Medical Sciences Kashan Iran; ^3^ Autoimmune Diseases Research Center Kashan University of Medical Sciences Kashan Iran; ^4^ Exir Azma Salam Iranian institute Research and Development Department Tehran Iran; ^5^ Department of Anatomy School of Medicine Shiraz University of Medical Sciences Shiraz Iran

**Keywords:** celiac disease, recurrent pneumonia, selective IgM deficiency

## Abstract

SIgMD is a rare immune disorder that occurs in a primary or secondary condition. Patients with recurrent infectious, cancers, and autoimmune disorders should be investigated to determine SIgMD.

## Graphical abstract

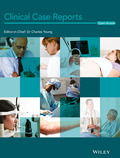

## 1 INTRODUCTION

Selective immunoglobulin M deficiency (SIgMD) is a rare immunodeficiency accompanied by some autoimmunity and cancers. In this study, we investigated a 58‐year‐old man with autoimmunity and recurrent pneumonia. SIgMD was determined by ESID criteria. Our case indicated that SIgMD should be evaluated in patients with autoimmunity and recurrent infections.

Selective IgM deficiency (SIgMD) as an uncommon immunodeficiency is defined by an isolated deficiency of serum IgM.^1^, ^2^ Other antibody levels and T‐cell immunity are usually normal.^2^ Patients with SIgMD respond normally to vaccination.^3^, ^4^ The prevalence of SIgMD is estimated about 0.03%‐1% of the population.^2^ Although the etiology of SIgMD is not well identified yet, several genetic and environmental factors have been proposed to its pathogenesis.^5^, ^6^, ^7^ Deletion in 22q11.2 chromosome is largely related to SIgMD, especially in subjects suffering from chronic otitis, developmental delay, velopharyngeal insufficiency, and dysmorphic features.^8^ This disorder is mainly divided into primary and secondary SIgMD. It may occur following other diseases including systemic lupus erythematosus (SLE) disease, Crohn's disease, multiple myositis disease, Hashimoto's disease, celiac disease, hematological malignancy, nephrotic syndrome, trauma, and protein‐losing enteropathy.^5^, ^9^, ^10^, ^11^, ^12^, ^13^, ^14^

Celiac disease is an autoimmune type of gastrointestinal disorder in which the immune system responds to foods with gluten through damaging the small intestine.^15^ Some reports have revealed that IgMD may be correlated to celiac disease. There are several studies pointing the prevalence of SIgMID in adult patients with celiac disease.^16^, ^17^

Recurrent bacterial and viral infections are the most common clinical symptoms of patients with SIgMD.^18^ This disorder enhances the risk of developing serious infections of polysaccharide‐containing organisms which lead to recurrent respiratory tract infections, urinary tract infections, recurrent sepsis, and diarrhea.^16^

To date, several SIgMD cases have been reported in Iran^2^; however, there is no study showing SIgMD in the Iranian patients with celiac disease and recurrent pneumonia. Hence, we reported a case of SIgMD in a patient suffering from celiac disease and recurrent pneumonia.

## 2 CASE HISTORY

Our case was a 58‐year‐old Iranian man referred to Shahid Beheshti hospital, Kashan, Iran. He suffered from chest pain, abdominal discomfort, and recurrent pneumonia. He had no family history of immunodeficiency diseases. His parents had no consanguinity and hereditary disease. Moreover, there was no health problem in his siblings. Our patient experienced recurrent pneumonia (four times/year) at age 14 years. Blood and throat swab cultures were performed for patient to diagnosis bacterial sepsis and infection cause(s). He was discharged from Kashan Shahid Beheshti hospital after a recovery period. In his 50th year, he suffered from chest discomfort and was referred to the hospital. There was not any sign of heart disease, and he was treated with palliative treatments. After three years, the patient experienced the first anemia accompanied by the elevated liver enzymes, dyspnea, and productive coughs. Thus, he was admitted to Kashan Shahid Beheshti hospital for investigating the cause(s) of these complications (Table 1).

**Table 1:** Laboratory characteristics of patients

	Cell number or Value	Total counted cells or normal range
Hematology		
WBC	6.15 × 10^3^/µL	10 × 10^3^/µL
Neutrophil	42.4%	10 × 10^3^/µL
Lymphocyte	45.4%	10 × 10^3^/µL
Monocyte	10.1%	10 × 10^3^/µL
Eosinophil	1.3%	10 × 10^3^/µL
Basophil	0.8%	10 × 10^3^/µL
RBC	4.17 × 10^6^/µL	50 × 10^4^/µL
Platelet	12.7 × 10^4^/µL	50 × 10^4^/µL
MCHC	28.8	
MCH	20.8	
RDW	15.3	
MCV	72.4	
Hemoglobin	9 g/dL	12‐14 g/dL
ESR	12 mm/h	Male up to 12 mm/h
Folic Acid	1.2	1.5‐17
Ferritin	3.4 ng/dL	Male: 16‐200
Biochemistry		
Bilirubin total	0.5 mg/dL	0.1 to 1.5 mg/dL
SGOT	56.5 U/L	Up to 42 U/L
SGPT	59.5 U/L	Up to 36 U/L
Alkaline phosphates	310	65‐300
Na	138 mEq/L	
K	3.4 mEq/L	
Cl	105 mEq/L	
FBS	85	70‐115
BUN	14 mg/dL	8‐25 mg/dL
Cr	0.8 mg/dL	0.4‐1.5 mg/dL
Cholesterol	147 mg/dL	150‐250 mg/dL
Triglyceride	67 mg/dL	60‐200 mg/dL
TSH	4 µ/mL	0.3‐5 µ/mL
FT4	10 Pm	10‐23 Pm
Serum protein electrophoresis		
Albumin	54 %	52%‐62%
Alpha 1. globulin	3.7%	2%‐6%
Alpha 2. globulin	10.5 %	8%‐13%
Beta globulin	14.9%	13%‐19%
Gamma globulin	19.8%	15%‐23%
Immunology		
IgM	30 mg/dL	56‐352 mg/dL
IgA	250 mg/dL	70‐312 mg/dL
IgE	3.2 mg/dL	1.53‐11.4 mg/dL
IgG	1076 mg/dL	630‐1349 mg/dL
IgG2‐subclass	369 mg/dL	60‐790 mg/dL
Measles specific IgG	120 mg/dL	80‐450 mg/dL
Mumps specific IgG	103 mg/dL	70‐500 mg/dL
Rubeola specific IgG	96 mg/dL	65‐460 mg/dL
CH50	140	70‐150
C3	128 mg/dL	88‐201 mg/dL
C4	32 mg/dL	16‐47 mg/dL
ANA	1.60	Positive: 1.0
Anti‐dsDNA antibody	Negative	
ANCA	Negative	
Rheumatoid factor	Negative	
Anti‐Smith	Negative	
Anti‐RNP	Negative	
HBS Ag	Negative	
HCV Ab	Negative	
tTG‐ IgA	347 U/mL	Neg < 10 Pos > 10
tTG‐ IgG	253 U/mL	Neg < 10 Pos > 10
Alpha 1 anti‐trypsin	242 ng/dL	100‐300 ng/dL
Anti‐mitochondrial	4.2 ng/dL	Up to 15 ng/dL
Anti‐smooth muscle	Negative	
CRP	Negative	
PSA	1.7 ng/dL	Up to 4 ng/dL
Carcinoembryonic antigen (CEA)	3.2 ng/mL	Smoker: <5.0 ng/mL
Lymphocyte markers		
CD2	88.5%	71%‐91%
CD3	57.74%	55%‐83%
CD4	34.66%	28%‐57%
CD8a	27.80%	10%‐39%
CD20	4.7 %	5%‐25%
CD19	6.08%	6%‐19%

Abbreviations: BUN, Blood urea nitrogen; FBS, Fasting blood sugar; PSA, Prostatic specific antigen; tTG‐IgA, anti‐tissue transglutaminase IgA.

**FIGURE 1:**
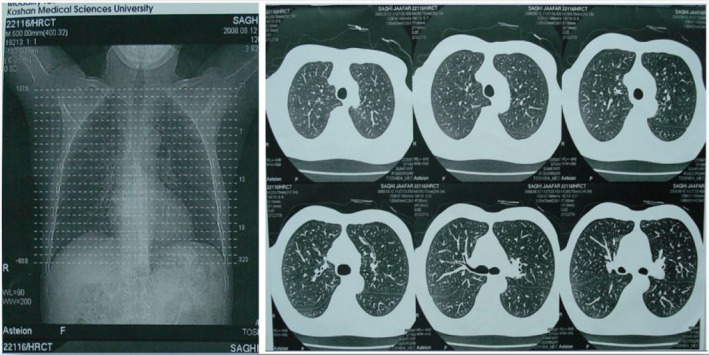
The thorax HRCT scan: No evidence of mass lesions and airway diseases were observed in the HRCT scan

Our case mentioned that he had history of tobacco smoking (20 pack‐years). Regarding that he was a smoker and suffered from anemia and weakness, body aches, fatigue, and episodic coughs, high resolution computed tomography (HRCT) scan and X‐ray (CXR) image of his thorax were performed. The result of CXR was normal. HRCT scan revealed that pulmonary parenchyma and its bronchial tree were normal. Furthermore, there was no evidence of abnormal air trapping, mass lesion, bronchiectasis, hyperaeration, and any airway diseases. Major mediastinal structures were also normal (Figure 1). However, the spirogram showed an obstructive pattern, due perhaps to chronic obstructive pulmonary disease (COPD). No significant change was observed in lung capacity upon the use of a bronchodilator. This finding suggested that chronic cough was associated with COPD.

**FIGURE 2:**
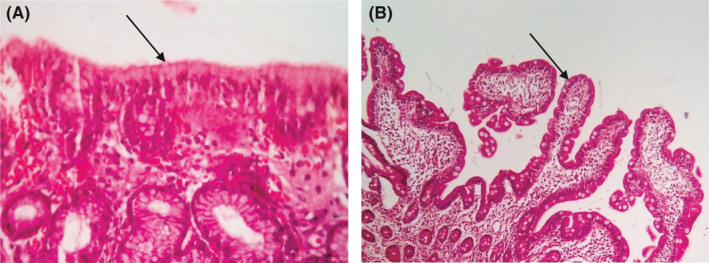
Endoscopic biopsies of patient. A, Initial biopsy of duodenal mucosa revealed atrophic lesions (Marsh 3C) characterized by total villous atrophy and numerous intraepithelial lymphocytes with hyperplastic granular components (10×). B, Duodenal mucosa was improved in the appearance of villi after gluten exclusion from the diet (40×)

Gastrointestinal specialists suggested that electrophoresis of serum proteins may be helpful to determine the actual cause(s) of the complications through excluding protein‐losing enteropathies, gammopathies, and autoimmune hepatitis. However, this method failed to clarify causative factors of these problems (Table 1). Therefore, the patient was discharged without any definite diagnosis. At age 57 years, some new clinical manifestations were observed in the patient including blistering skin lesions and abdominal pain triggered by eating the bread. His cough and dyspnea were not accompanied by blistering lesions and abdominal pain. Skin lesions were evaluated by dermatologists. It was reported that our case had dermatitis herpetiformis which is considered as an extra‐intestinal manifestations of celiac disease.^19^ Therefore, the patient was suspected to have this disease. Anti‐tissue transglutaminase IgA (tTG‐IgA) and anti‐tissue transglutaminase IgG (tTG‐ IgG) antibodies as celiac disease markers were measured by an enzyme‐linked immunosorbent assay (ELISA) kit according to the manufacturer's protocol (Mabtech, Sweden). Our case had the increased values of tTG‐IgA and tTG‐IgG antibodies (Table 1). Additional confirmation of celiac disease diagnosis was provided by endoscopic biopsies of gastric and duodenum. Pathology reports revealed celiac disease and acute gastritis due to helicobacter pylori. The results of pathology investigations indicated total villous atrophy (atrophic lesions, Marsh 3C) characterized by the total absence of villi and numerous intraepithelial lymphocytes containing hyperplastic granular components (Figure 2A). Celiac disease was also approved by subsiding disease symptoms after gluten‐exclusion diet (Figure 2). However, a gluten‐free diet failed to reduce lung infections. Fiberoptic bronchoscopy was used to explain causative agent(s) of recurrent pneumonia and cough. The results of bronchoscopy revealed congenital dysplasia of the right lobe of the lung. The patients were investigated according to the European Society for Immunodeficiency (ESID) criteria.^4^ In addition to complement components, the levels of total IgM, IgA, IgG, IgE, and other antibodies were studied using an ELISA kit according to the manufacturer's protocol (Mabtech, Sweden). Furthermore, the frequencies of CD3, CD4, CD8a, CD19, and CD20 as lymphocyte markers were measured to determine the defects in B‐cell and T‐cell immunity. The percentages of CD markers were determined by a FACSCalibur flow cytometer (Becton Dickinson) and primary monoclonal antibodies (Table 2). Moreover, our case received measles, mumps, and rubella (MMR) vaccine and specific IgG antibodies were measured using ELISA. The data of this study revealed that patient had the reduced values of IgM and CD20 marker as a known B‐cell marker (Table 1). Moreover, anti‐nucleotide antibody (ANA) showed the increased level in comparison with normal range (Table 1). However, there were no antibodies against diagnostic markers of SLE and rheumatoid arthritis diseases (Table 1). Other results of immunologic, hematologic, and biochemistry tests are shown in Table 1.

**Table 2:** **Antibodies used for the determination** of **immune situatuin of the patient by flow cytometry**

Fluorochrome/antibody	Isotype	Company (all from USA)
FITC anti‐human CD19	Mouse IgG1, κ	BioLegend
FITC anti‐human CD20	Mouse IgG2b, κ	BioLegend
FITC anti‐human CD4	Mouse IgG1, κ	BioLegend
FITC anti‐human CD8a	Mouse IgG1, κ	BioLegend
FITC anti‐human CD3	Mouse IgG1, κ	BioLegend

Our data revealed that MMR‐vaccine‐induced antibodies were detectable after 1 month (Table 1). The results of immunologic tests and MMR‐specific IgG responses demonstrated that patient might have a rare immune disorder, called SIgMD. To determine the correlation of SIgMD with celiac disease, the gluten was excluded from the diet. After 1 year, the immunologic tests were rechecked. The same trend was observed in the level of IgM (Table 3). This observation suggested that the gluten‐free diet did not affect the serum IgM level in patient. It is thought that recurrent lung infections were associated with SIgMD.

**Table 3:** The levels of antibodies after gluten exclusion from the diet

Antibody	Value	Normal range
IgM	34 mg/dL	56‐352 mg/dL
IgA	137 mg/dL	70‐312 mg/dL
IgE	4.8 mg/dL	1.53‐11.4 mg/dL
IgG	1298 mg/dL	630‐1349 mg/dL

## 3 DISCUSSION

SIgMD is a rare form of immunodeficiency which may relate to some health problems such as cancer, autoimmunity, allergic disorders, and gastrointestinal diseases.^2^, ^18^ This disorder can be asymptomatic or with various viral and bacterial infections.^20^ Thus, patients suffering from different infections and health problems should be evaluated to clarify SIgMD. In the present study, we investigated the IgM level in the patient who had recurrent hospital admission due to chest and abdominal pains, repetitive pneumonia, and anemia.

Some clinical infectious presentations of SIgMD include recurrent otitis media, pneumonia, chronic sinusitis, bronchiectasis, sepsis, meningitis, urinary tract infections, and cellulitis.^18^ Some of the frequent microbial organisms are *Streptococcus pneumoniae, Hemophilus influenza, Pseudomonas aeruginosa, Neisseria meningitides,* and *Aspergillus fumigates*.^12^, ^21^ Our microbiology workup revealed that *Streptococcus pneumonia*e was the main cause of recurrent pneumonia. In regard to the relationship of SIgMD with susceptibility to recurrent infections, natural IgM recognizes epitopes of phosphorylcholine expressed on the cell walls of many organisms involved in various infections and thereby reducing the risk of developing recurrent infections in healthy subjects.^18^ Another mechanism in increased susceptibility to infections in SIgMD may associate with impaired IgG‐specific antibody response to T‐independent polysaccharide antigens.^9^, ^18^, ^22^, ^23^

Celiac disease is characterized by great damage of the small intestinal mucosa, loss of absorptive villi, and hyperplasia of the crypts, leading to malabsorption, especially folic acid, and anemia.^24^, ^25^ In this study, we found that our case had the reduced values of folic acid, ferritin, and hemoglobin. These findings along with previous reports suggest that anemia in the patient may result in celiac disease.^25^, ^26^

Erythema multiform is a skin condition caused by a hypersensitivity reaction to infections and drugs.^27^ Some reports pointing to this reactive mucocutaneous disorder as a skin lesion of celiac disease.^15^ Our patient also complained of dermatitis herpetiformis. This observation consistent with other evidence suggests that skin lesions in patients with celiac disease were associated with viral and bacterial infections due to SIgMD.^18^

Previous reports have shown that celiac symptoms subsided after excluding gluten from the diet.^28^, ^29^ In the present study, celiac disease was confirmed by endoscopic biopsies and its symptoms were disappeared upon gluten exclusion from the diet, however the IgM level in the patient did not show any significant change. Several studies revealed the incidence of IgMD in patients with celiac disease.^14^, ^16^, ^30^, ^31^ In line with notion, IgMD may be associated with celiac disease, due perhaps to lymphoreticular dysfunction which is stimulated by the exposure to gluten antigen and/or the defect in the differentiation of B cells into IgM‐immunoglobulin‐secreting cells.^32^, ^33^ It is reported that a complete restoration of normal IgM value occurred following treatment with a gluten‐free diet.^17^ However, we observed no notable change in IgM titer upon gluten exclusion from the diet. Therefore, our case was the first report to show that SIgMD may not associate with celiac disease. This observation proposes that our patient may have symptomatic primary SIgMD accompanied by celiac disease. Nonetheless, genetic analyses should be employed to explore primary SIgMD in patient with celiac disease.

## 4 CONCLUSION

Selective immunoglobulin M deficiency is a rare immune disorder that can occur in a primary or secondary condition in patients with recurrent infections, autoimmunity, and cancers. Primary SIgMD can affect both children and adults. Patients suffering from recurrent infectious diseases, cancers, and autoimmune disorders should be investigated by ESID criteria to determine possible defect(s) in the immune system, especially SIgMD.

## CONFLICT OF INTEREST

None declared.

## AUTHOR CONTRIBUTIONS

MHA: participated in the diagnosis of SIgMD. MR: as a gastrointestinal specialist: participated in the diagnosis of celiac disease and its treatment. RA: carried out some of the experiments. HM: drafted the manuscript and participated in the study design. All authors: read and approved the final manuscript.

## ETHICAL APPROVAL

This study was approved by the Ethics Committee of Kashan University of Medical Science.

## Data Availability

All data generated or analyzed during this study are included in this published case report.
